# The cool things to know about TRPM8!

**DOI:** 10.1080/19336950.2020.1841419

**Published:** 2020-11-04

**Authors:** Mircea Iftinca, Christophe Altier

**Affiliations:** Department of Physiology and Pharmacology, Inflammation Research Network-Snyder Institute for Chronic Diseases and Alberta Children’s Hospital Research Institute, Cumming School of Medicine, University of Calgary, Calgary, Alberta, Canada

**Keywords:** Transient receptor potential channel subfamily M (melastatin) member 8 (TRPM8), PIP_2_, protein kinase C (PKC), G proteins

## Abstract

Transient receptor potential melastatin 8 (TRPM8) channels play a central role in the detection of environmental cold temperatures in the somatosensory system. TRPM8 is found in a subset of unmyelinated (C-type) afferents located in the dorsal root (DRG) and trigeminal ganglion (TG). Cold hypersensitivity is a common symptom of neuropathic pain conditions caused by cancer therapy, spinal cord injury, viral infection, multiple sclerosis, diabetes, or withdrawal symptoms associated with chronic morphine treatment. Therefore, TRPM8 has received great attention as a therapeutic target. However, as the activity of TRPM8 is unique in sensing cool temperature as well as warming, it is critical to understand the signaling transduction pathways that control modality-specific activity of TRPM8 in healthy versus pathological settings. This review summarizes recent advances in our understanding of the mechanisms involved in the regulation of the TRPM8 activity.

## Introduction

More than 50 years have passed since the first recording of the transient receptor potential (TRP) channel current in *Drosophila* in which the mutant channel generated a photoreceptor light response that decayed to baseline during prolonged light illumination [1]. Almost exactly 20 years later the mutant gene responsible for this phenomenon was identified and termed *trp* [2]. Since then, the complete identification and characterization of the large family of TRP channels have been completed, including the TRPC1 in the mid 1990 [3,4], followed by the vanilloid receptor TRPV1 [5], the camphor receptor TRPV3 [6] and, much later, the menthol receptor TRPM8 [7,8]. The TRPM8 channel was first discovered in mouse sensory dorsal root ganglia (DRG) neurons [9], although numerous TRPM8 isoforms have now been described in various mammalian tissues such as prostate, cardiovascular system, bronchopulmonary tissue, bladder and urogenital tract and many others [10]. This channel is expressed in peripheral sensory neurons of small size (unmyelinated C fibers) to transduce environmental cold stimuli [7,11,12]. Notably, its ability to sense cold is finely tuned in various species for better ambient temperature adaptation [13] and by taking part in the energy metabolism [14,15]. In a recent study it has even been proposed that, along with TRPA1 and TRPV1 channels, TRPM8 underlies all thermosensation in mice [16]. Indeed, genetic manipulation of TRPM8 in mice leads to a loss of environmental cold sensation [11,17,18] and even the ability to detect warm [19]. In humans reduced TRPM8 expression leads to attenuated cold and pain sensation [20]. Conversely, hyperactivity of the channel plays a central role in disease pain states such as cold allodynia after inflammation or nerve injury [21,22]. A growing number of evidence link this channel to various tumors, such as prostate, pancreas, colon, breast, lung, and skin [23]. In addition, TRPM8 has been linked to migraine [24] but also to dry eye disease [25], irritable bowel syndrome, oropharyngeal dysphagia, chronic cough, or hypertension [26–29] and the list continues to grow.

It is, therefore, not surprising that attention to TRPM8 activity modulation has been getting lately by numerous research groups and pharmaceutical companies although, oftentimes, their results are conflicting. This review is not aimed at compiling all knowledge gathered to date regarding modulation of the TRPM8 activity but rather at updating and summarizing the new research results obtained in the last few years.

## TRPM8 channel

The TRPM8 channel is a voltage-gated, nonselective cation channel that contains six transmembrane segments (S1-S6) and large intracellular amino and carboxyl termini [30,31]. The S2 and S3 segments possess the binding sites for the cooling agents: menthol and icillin, respectively. The S4 segment and the region between S4 and S5 carry out the function of voltage sensing while the region between S5 and S6 forms the channel pore [30,31]. Like many TRP channels, TRPM8 has polymodal, distinct gating mechanisms, being activated by innocuous to noxious cool temperatures (10–28° C), membrane depolarization, cooling agents such as menthol and icilin and changes in osmolality and pH [7,8,11,12,32–37]. Most sensory modalities undergo calcium-dependent desensitization in which the activity of the channel decreases despite the continuous presence of the stimulus [7,38,39]. Indeed, removing the extracellular calcium markedly reduces desensitization [7]. Recently, Diver et al. provided insight into the structural basis of the desensitization process [40]. The authors showed the critical contribution of five negatively charged residues located in the S2 and S3 transmembrane helices and within the S2-S3 linker. They proposed a new model in which the channel had only two distinct conformational states, closed and desensitized, and upon activation does not undergo dramatic conformational changes within the S3-S4 transmembrane segments but rather only in the outer region of the pore.

## TRPM8 modulation by PIP_2_

Phosphoinositides are well-known modulators of a large number of ion channels at the plasma membrane. In the early 2000, B. Hille’s group reported that recovery from muscarinic-induced inhibition of the M current was phosphatidylinositol 4,5-bisphosphate (PIP_2_) dependent. His lab and others later established that PIP2 was necessary to maintain the function of potassium-selective channels, including voltage-gated potassium (Kv) channels, inwardly-rectifying potassium (Kir) channels, and calcium-activated potassium (KCa) [41].

While TRP channels exhibit poor voltage sensitivity they have a tetrameric architecture similar to that of Kv channels. Accordingly, PIP2 has been shown to positively modulate the function of 20 out of 28 channels in the TRP superfamily, including TRPM8 [42]. Daniels *et al*. also reported that PIP_2_ depletion could shift the channel voltage dependence toward more positive potentials, reducing the cold- and menthol-activated currents [38]. Later, another study showed that warm ambient temperatures (40°C) shift the channel temperature threshold through a calcium-independent mechanism involving PIP_2_ [43]. The locus of PIP_2_ binding has been under controversy until recently when it was shown, in the cryoEM structure of TRPM8, that PIP_2_ binds in a pocket cradled by the pre-S1 elbow and S1 helix of one subunit and the S5 helix of the adjacent subunit [40,44].

Activation of Gq-coupled receptors stimulates the calcium-sensitive phospholipase Cβ (PLCβ) that cleaves PIP2 into soluble inositol 1,4,5-trisphosphate (IP3) and membrane-bound diacylglycerol. Therefore, the opening of TRPM8 channels leads to PLCβ activation and PIP2 hydrolysis which activates TRPM8 and acts as a positive modulator of cold and menthol stimuli whereas PIP_2_ depletion inhibits channel activity [7,38,39,45,46]. While PLCβ received attention in the modulation of TRPM8, the genetic manipulation of PLCδ4 leads to an increase in cold- and menthol-induced current in DRG neurons [47], suggesting that other PLC isoforms that contain a calcium-binding motif could be involved in this process.

Another mechanism involved in PIP_2_-dependent regulation of TRPM8 activity has emerged lately. Phosphoinositide-interacting regulator of TRP (PIRT), a two transmembrane domain protein specifically expressed in the DRG, trigeminal ganglia, and enteric neurons of the peripheral nervous system, was first identified as a positive modulator of TRPV1 via PIP_2_ interaction [48]. PIRT was then found to increase voltage dependence and sensitivity to menthol of the TRPM8 channel by having a synergistic effect with PIP_2_ [49]. This regulatory mechanism was suggested to result from an increase in the local concentration of PIP2 in complex with TRPM8 and PIRT [50]. However, the effect of PIRT appeared to be species specific since Hilton *et al*. found that while mouse PIRT enhanced mouse TRPM8 activity, human PIRT attenuated TRPM8 conductance [51]. This distinct regulatory mechanism provides real physiological significance in terms of species-specific fine-tuning of thermosensation.

## TRPM8 modulation by G proteins

Pain and heat are the two main cardinal symptoms of inflammation. Previous work revealed that inflammatory mediators, including BK and histamine, inhibited TRPM8 channel function, a process that could be implicated in core body temperature regulation.

While BK and histamine are known to act on Gαq-coupled receptors, several downstream signaling pathways have been proposed to promote channel inhibition.

Since PIP2 binding to TRPM8 maintains channel function, it was originally suggested that activation of the bradykinin B2 receptor could reduce TRPM8 activity via PLCβ activation and PIP_2_ depletion [52]. Nevertheless, the mechanism by which G protein-coupled receptors (GPCRs) modulate TRPM8 has remained controversial over the years. Two studies made the surprising observation that GPCRs coupled to Gs protein and activation of PKA were able to inhibit TRPM8 channels [53,54]. Moreover, clonidine activation of the α2 adrenergic receptor, which signals through Gi proteins and thus inhibit PKA activity, was also reported to inhibit TRPM8 [55] although another study found no effect of activating this pathway on TRPM8 inhibition in DRG neurons [56]. A later publication from Peter McNaughton’s lab challenged the idea of an involvement of PIP2 degradation in mediating channel inhibition. They proposed that, upon bradykinin receptor activation, Gαq could directly bind to the channel and inhibit its activity evoked by cold or menthol [57,58]. This finding was observed using recombinant B2 receptor and TRPM8 channel in HEK cells as well as on native TRPM8 current recorded from isolated DRG neurons. To add to the complexity of TRPM8 regulation by G protein-coupled receptors, another study showed that stimulation of TRPM8 alone was able to mediate a metabotropic signaling via Gαq and calcium release from internal stores [59]. Liu et al. recently proposed an integrated mechanism in which Gαq enhances channel sensitivity to PIP2 levels [60] whereas another study, using DRG neurons and genetically engineered mice supported the sole role for Gαq in inhibiting TRPM8 following bradykinin receptor activation [61]. Therefore, it is still unclear to what extend TRPM8 function is controlled by Gαq and if the interaction of the channel with the G protein is able to compete with Gαq-coupled receptor activation.

## Regulation by phosphorylation

Several signaling cascades are involved in the regulation of TRPM8 activity through phosphorylation. Activation of PLCβ leads to not only the reduction of PIP2 levels but also to the activation of protein kinase C (PKC) that phosphorylates a large number of TRP channels including TRPM8. Various PKC isoforms (ε, α, and β) are present in the small unmyelinated C fibers, including the TRPM8-expressing subpopulation [62,63]. Previous studies showed that activation of PKC produced menthol-induced desensitization of TRPM8 as well as dephosphorylation and downregulation of channel expression in expression systems and rat neonatal DRG neurons [64–66]. However, none of the nine putative PKC phosphorylation sites of TRPM8 were involved in channel modulation and the authors concluded that PKC had an indirect effect on menthol-induced desensitization [65]. Activation of PKC did not increase the phosphorylation state of the channel but rather activated the calcium and calmodulin-dependent serine/threonine protein phosphatase calcineurin, indicating a dephosphorylation-induced desensitization process. Furthermore, PKC inhibitors failed to block desensitization [39] and menthol-induced TRPM8 current [45]. Recently, our group described a crosstalk relationship between TRPM8 and PKC in the context of opioid-induced cold sensitivity. Indeed, postoperative shivering and cold hypersensitivity are major side effects of acute and chronic opioid treatments, respectively. Our findings showed that activation of the mu-opioid receptor by morphine induced a PKCβ-dependent inhibition of TRPM8 desensitization (disinhibition) in response to cold or menthol [67]. The loss of TRPM8 desensitization was prevented by the use of a selective PKCβ blocker. We then identified a new PKC phosphorylation site in the carboxy terminus region located in the proximity of the S6-TRP box linker. The TRP box acts as a central determinant of channel gating evoked by voltage, menthol and cold and has several PIP2 binding sites that control channel gating [30,31,36,68]. Site-directed mutagenesis of this consensus site was able to abrogate the morphine-induced inhibition of TRPM8 desensitization.

Another kinase previously described for regulating TRPM8 is the protein kinase A (PKA). Previous studies showed that PKA activation inhibits TRPM8 channel in expression systems as well as DRG neurons [53,54]. In contrast, inhibition of PKA, as a result of α2 adrenergic receptors activation, was found to reduce TRPM8 activity in menthol-sensitive DRG neurons [55]. Using HEK cells heterologously expressing TRPM8, Pabbidi et al. found that Phorbol 12,13-Dibutyrate (PDBu), a PKC activator, and forskolin, a PKA activator, both downregulated TRPM8-mediated currents [66]. Of note, TRPM8 was found to in turn regulate PKA activity. In a recent study, PC12 cells exposed to oxygen-glucose deprivation were less susceptible to become apoptotic when TRPM8 was blocked. This antiapoptotic effect observed upon TRPM8 inhibition was mediated by cAMP production and PKA activation [69].

Much attention has been given to the contribution of TRPM8 in cancer, and particularly prostatic cancer [23]. Recent findings have shown that TRPM8 protein is a receptor for testosterone which directly binds to and promotes channel opening [70,71]. At picomolar concentrations, the application of testosterone induces full activation of the TRPM8 and interestingly mediates a cooling sensation in human skin [70]. In addition, testosterone can regulate the expression of the *TRPM8* gene through the androgen response element [70–72]. Still, controversy surrounds the mechanisms by which testosterone regulates TRPM8-mediated cold perception. A recent study reported the inhibitory effect of testosterone on TRPM8. At nanomolar concentrations, testosterone suppressed TRPM8-mediated currents in native dorsal root ganglion (DRG) neurons and HEK cells co-expressing recombinant TRPM8 and the androgen receptor, but not TRPM8 alone. The study identified a non-canonical signaling pathway downstream of the androgen receptor (AR) activation that involved Src, ERK, and Akt [73]. In contrast to earlier results, testosterone had no effect on TRPM8 in DRG neurons from AR null mice.

The tyrosine kinase Src plays a major role in cancer, neuronal growth, and synaptic plasticity [74]. Src is activated by testosterone and numerous inflammatory mediators or growth factors acting on tyrosine kinase receptors. As such, activation of the Nerve Growth Factor (NGF) receptor, TrkA, was first reported to induce an increase in the cold sensitivity of cultured rat DRG neurons [75]. In contrast to this study, Manolache *et al*. found only a modest effect of NGF on channel phosphorylation. However, they showed that TRPM8 was constitutively tyrosine phosphorylated by Src in heterologous expression systems or DRGs. Pharmacological or genetic inhibition of Src reduced both TRPM8 tyrosine phosphorylation and cold-induced channel activation [76].

Lastly, TRP channel-associated factors, TCAF1 and TCAF2, have been shown to regulate TRPM8 channels in the prostate, with opposing effects [77]. In single-channel recordings, TCAF1 expression was able to increase TRPM8 channel trafficking to the membrane as well as channel gating. In contrast, TCAF2 completely silenced it. Importantly, this was shown to be achieved through PI3K phosphorylation of the channel as the use of wortmannin reversed this effect [77].

## Conclusions

**Figure 1.**
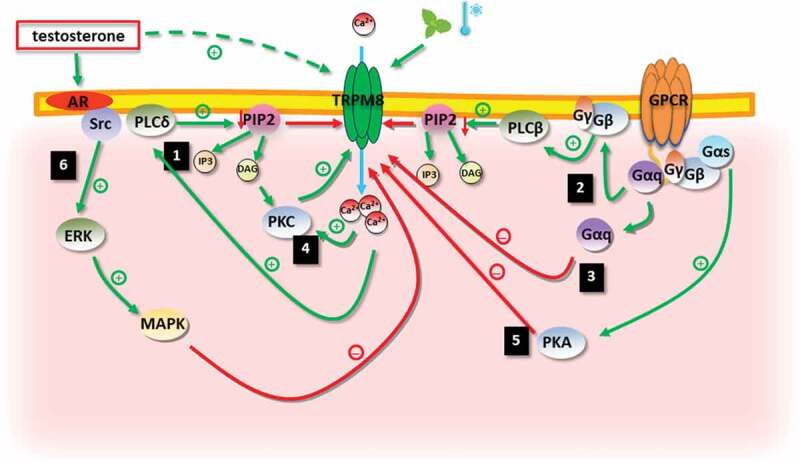
Identified signaling pathways involved in TRPM8 channel regulation. 1 – Activation of PLCδ4 by calcium influx leads to PIP2 depletion and inhibition of TRPM8; 2 – Gq-coupled receptor-mediated stimulation of PLCβ leads to PIP2 depletion and inhibition of TRPM8. 3 – In response to the stimulation of the bradykinin receptor (B2R), Gαq directly binds to and inhibit TRPM8; 4 – DAG and calcium influx mediate the activation of PKC, which contributes to a Ca2+ mediated desensitization of TRPM8; 5 – Activation of Gs-coupled receptors leads to a PKA-mediated inhibition of TRPM8; 6 – Testosterone activates TRPM8 directly or indirectly through the AR signaling pathway that involves Src, ERK and MAPK. Abbreviations: AR-androgen receptor, DAG – diacylglycerol, ERK – extracellular signal-regulated kinase, GPCR – G protein-coupled receptor, IP3 – inositol trisphosphate, MAPK – mitogen-activated protein kinase, PIP2 – phosphatidylinositol bisphosphate, PKA – protein kinase A, PKC – protein kinase C, PLC-phospholipase C

Although the role of TRPM8 channel in sensing environmental mild cold temperatures is well established, the knowledge regarding its regulation in health and diseases is still growing and controversial. Here we have reviewed recent findings on the regulation of the TRPM8 channel by phosphoinositide, G proteins and protein kinases, in heterologous expression systems, isolated DRG neurons, and animal studies (Figure 1).

The described molecular mechanisms will certainly have to be validated in larger animals and humans, particularly because of the species-specific sequence variation of TRPM8 that has been developed for thermal adaptation through evolution [13].

Clearly, new advances in knowledge regarding the physiological and pathological roles of TRPM8 along with the characterization of the natural ligands of the channel will guide researchers toward a better understanding of its regulatory mechanisms.

In fact, a recent study has proposed a role for TRPM8 activation in limiting the severity of human enterovirus, Coxsackievirus B virus infection [78]. Another one discovered a short TRPM8 isoform, termed 4TM-TRPM8, in mitochondria-associated endoplasmic reticulum membrane of keratinocytes and prostate epithelial cells [79]. Therefore, a better comprehension of TRPM8 function in thermosensation, pathogen infection, or cancer, and a deeper characterization of its expression in organs and cell types, will help to identify the relevant signaling pathways that can modulate TRPM8.

## References

[1] Cosens DJ, Manning A. Abnormal electroretinogram from a Drosophila mutant. Nature. 1969;224(5216):285–287. 5344615 10.1038/224285a0

[2] Montell C, Rubin GM. Molecular characterization of the Drosophila trp locus: a putative integral membrane protein required for phototransduction. Neuron. 1989;2(4):1313–1323. 2516726 10.1016/0896-6273(89)90069-x

[3] Wes PD, Chevesich J, Jeromin A, et al. TRPC1, a human homolog of a Drosophila store-operated channel. Proc Natl Acad Sci U S A. 1995;92(21):9652–9656. 7568191 10.1073/pnas.92.21.9652 PMC40860

[4] Zhu X, Chu PB, Peyton M, et al. Molecular cloning of a widely expressed human homologue for the Drosophila trp gene. FEBS Lett. 1995;373(3):193–198. 7589464 10.1016/0014-5793(95)01038-g

[5] Caterina MJ, Schumacher MA, Tominaga M, et al. The capsaicin receptor: a heat-activated ion channel in the pain pathway. Nature. 1997;389(6653):816–824. 9349813 10.1038/39807

[6] Peier AM, Reeve AJ, Andersson DA, et al. A heat-sensitive TRP channel expressed in keratinocytes. Science. 2002;296(5575):2046–2049. 12016205 10.1126/science.1073140

[7] McKemy DD, Neuhausser WM, Julius D. Identification of a cold receptor reveals a general role for TRP channels in thermosensation. Nature. 2002;416(6876):52–58. 11882888 10.1038/nature719

[8] Peier AM, Moqrich A, Hergarden AC, et al. A TRP channel that senses cold stimuli and menthol. Cell. 2002;108(5):705–715. 11893340 10.1016/s0092-8674(02)00652-9

[9] Reid G, Flonta ML. Physiology. Cold current in thermoreceptive neurons. Nature. 2001;413(6855):480. 11586349 10.1038/35097164

[10] Blanquart S, Borowiec AS, Delcourt P, et al. Evolution of the human cold/menthol receptor, TRPM8. Mol Phylogenet Evol. 2019;136:104–118. 30980935 10.1016/j.ympev.2019.04.011

[11] Bautista DM, Siemens J, Glazer JM, et al. The menthol receptor TRPM8 is the principal detector of environmental cold. Nature. 2007;448(7150):204–208. 17538622 10.1038/nature05910

[12] McKemy DD. How cold is it? TRPM8 and TRPA1 in the molecular logic of cold sensation. Mol Pain. 2005;1:16. 15847696 10.1186/1744-8069-1-16 PMC1087877

[13] Yang S, Lu X, Wang Y, et al. A paradigm of thermal adaptation in penguins and elephants by tuning cold activation in TRPM8. Proc Natl Acad Sci U S A. 2020;117(15):8633–8638. 32220960 10.1073/pnas.1922714117 PMC7165450

[14] Senaris R, Ordas P, Reimundez A, et al. Mammalian cold TRP channels: impact on thermoregulation and energy homeostasis. Pflugers Arch. 2018;470(5):761–777. 29700598 10.1007/s00424-018-2145-9

[15] Uchida K, Dezaki K, Yoneshiro T, et al. Involvement of thermosensitive TRP channels in energy metabolism. JPS. 2017;67(5):549–560. 28656459 10.1007/s12576-017-0552-x PMC10717017

[16] Yarmolinsky DA, Peng Y, Pogorzala LA, et al. Coding and plasticity in the mammalian thermosensory system. Neuron. 2016;92(5):1079–1092. 27840000 10.1016/j.neuron.2016.10.021 PMC5145739

[17] Colburn RW, Lubin ML, Stone DJ Jr., et al. Attenuated cold sensitivity in TRPM8 null mice. Neuron. 2007;54(3):379–386. 17481392 10.1016/j.neuron.2007.04.017

[18] Dhaka A, Murray AN, Mathur J, et al. TRPM8 is required for cold sensation in mice. Neuron. 2007;54(3):371–378. 17481391 10.1016/j.neuron.2007.02.024

[19] Paricio-Montesinos R, Schwaller F, Udhayachandran A, et al. The sensory coding of warm perception. Neuron. 2020;106(5):830–841. 10.1016/j.neuron.2020.02.035 PMC7272120 32208171

[20] Gavva NR, Sandrock R, Arnold GE, et al. Reduced TRPM8 expression underpins reduced migraine risk and attenuated cold pain sensation in humans. Sci Rep. 2019;9(1):19655. 31873179 10.1038/s41598-019-56295-0 PMC6927963

[21] Proudfoot CJ, Garry EM, Cottrell DF, et al. Analgesia mediated by the TRPM8 cold receptor in chronic neuropathic pain. Curr Biol. 2006;16(16):1591–1605. 16920620 10.1016/j.cub.2006.07.061

[22] Su L, Wang C, Yu YH, et al. Role of TRPM8 in dorsal root ganglion in nerve injury-induced chronic pain. BMC Neurosci. 2011;12:120. 22111979 10.1186/1471-2202-12-120 PMC3235975

[23] Hantute-Ghesquier A, Haustrate A, Prevarskaya N, et al. TRPM Family Channels in Cancer. Pharmaceuticals (Basel). 2018;11:2. 10.3390/ph11020058 PMC6027338 29875336

[24] Rainero I, Roveta F, Vacca A, et al. Migraine pathways and the identification of novel therapeutic targets. Expert Opin Ther Targets. 2020;24(3):245–253. 32054351 10.1080/14728222.2020.1728255

[25] Yang JM, Wei ET, Kim SJ, et al. TRPM8 channels and dry eye. Pharmaceuticals (Basel). 2018;11:4. 10.3390/ph11040125 PMC6316058 30445735

[26] Alvarez-Berdugo D, Rofes L, Casamitjana JF, et al. TRPM8, ASIC1, and ASIC3 localization and expression in the human oropharynx. Neurogastroenterology Motil off J Eur Gastrointestinal Motil Soc. 2018;30(11):e13398. 10.1111/nmo.13398 29971861

[27] Bonvini SJ, Belvisi MG. Cough and airway disease: the role of ion channels. Pulm Pharmacol Ther. 2017;47:21–28. 28669932 10.1016/j.pupt.2017.06.009

[28] Henstrom M, Hadizadeh F, Beyder A, et al. TRPM8 polymorphisms associated with increased risk of IBS-C and IBS-M. Gut. 2017;66(9):1725–1727. 27974553 10.1136/gutjnl-2016-313346 PMC5561393

[29] Huang F, Ni M, Zhang JM, et al. TRPM8 downregulation by angiotensin II in vascular smooth muscle cells is involved in hypertension. Mol Med Rep. 2017;15(4):1900–1908. 28138709 10.3892/mmr.2017.6158

[30] Bidaux G, Sgobba M, Lemonnier L, et al. Functional and modeling studies of the transmembrane region of the TRPM8 channel. Biophys J. 2015;109(9):1840–1851. 26536261 10.1016/j.bpj.2015.09.027 PMC4643257

[31] Yin Y, Wu M, Zubcevic L, et al. Structure of the cold- and menthol-sensing ion channel TRPM8. Science. 2018;359(6372):237–241. 29217583 10.1126/science.aan4325 PMC5810135

[32] Liu B, Fan L, Balakrishna S, et al. TRPM8 is the principal mediator of menthol-induced analgesia of acute and inflammatory pain. Pain. 2013;154(10):2169–2177. 23820004 10.1016/j.pain.2013.06.043 PMC3778045

[33] Macpherson LJ, Hwang SW, Miyamoto T, et al. More than cool: promiscuous relationships of menthol and other sensory compounds. Mol Cell Neurosci. 2006;32(4):335–343. 16829128 10.1016/j.mcn.2006.05.005

[34] Oz M, El Nebrisi EG, Yang KS, et al. Cellular and Molecular Targets of Menthol Actions. Front Pharmacol. 2017;8:472. 28769802 10.3389/fphar.2017.00472 PMC5513973

[35] Quallo T, Vastani N, Horridge E, et al. TRPM8 is a neuronal osmosensor that regulates eye blinking in mice. Nat Commun. 2015;6:7150. 25998021 10.1038/ncomms8150 PMC4455064

[36] Raddatz N, Castillo JP, Gonzalez C, et al. Temperature and voltage coupling to channel opening in transient receptor potential melastatin 8 (TRPM8). J Biol Chem. 2014;289(51):35438–35454. 25352597 10.1074/jbc.M114.612713 PMC4271229

[37] Andersson DA, Chase HW, Bevan S. TRPM8 activation by menthol, icilin, and cold is differentially modulated by intracellular pH. J Neurosci. 2004;24(23):5364–5369. 15190109 10.1523/JNEUROSCI.0890-04.2004 PMC6729305

[38] Daniels RL, Takashima Y, McKemy DD. Activity of the neuronal cold sensor TRPM8 is regulated by phospholipase C via the phospholipid phosphoinositol 4,5-bisphosphate. J Biol Chem. 2009;284(3):1570–1582. 19019830 10.1074/jbc.M807270200 PMC2615505

[39] Yudin Y, Lukacs V, Cao C, et al. Decrease in phosphatidylinositol 4,5-bisphosphate levels mediates desensitization of the cold sensor TRPM8 channels. J Physiol. 2011;589(Pt24):6007–6027. 22005680 10.1113/jphysiol.2011.220228 PMC3286682

[40] Diver MM, Cheng Y, Julius D. Structural insights into TRPM8 inhibition and desensitization. Science. 2019;365(6460):1434–1440. 31488702 10.1126/science.aax6672 PMC7262954

[41] Hille B, Dickson EJ, Kruse M, et al. Phosphoinositides regulate ion channels. Biochim Biophys Acta. 2015;1851(6):844–856. 25241941 10.1016/j.bbalip.2014.09.010 PMC4364932

[42] Rohacs T. Phosphoinositide regulation of TRP channels. Handb Exp Pharmacol. 2014;223:1143–1176. 24961984 10.1007/978-3-319-05161-1_18 PMC4527175

[43] Fujita F, Uchida K, Takaishi M, et al. Ambient temperature affects the temperature threshold for TRPM8 activation through interaction of phosphatidylinositol 4,5-bisphosphate. J Neurosci. 2013;33(14):6154–6159. 23554496 10.1523/JNEUROSCI.5672-12.2013 PMC6618937

[44] Yin Y, Le SC, Hsu AL, et al. Structural basis of cooling agent and lipid sensing by the cold-activated TRPM8 channel. Science. 2019;363:6430. 10.1126/science.aav9334 PMC6478609 30733385

[45] Sarria I, Ling J, Zhu MX, et al. TRPM8 acute desensitization is mediated by calmodulin and requires PIP(2): distinction from tachyphylaxis. J Neurophysiol. 2011;106(6):3056–3066. 21900509 10.1152/jn.00544.2011 PMC3234095

[46] Rohacs T, Lopes CM, Michailidis I, et al. PI(4,5)P2 regulates the activation and desensitization of TRPM8 channels through the TRP domain. Nat Neurosci. 2005;8(5):626–634. 15852009 10.1038/nn1451

[47] Yudin Y, Lutz B, Tao YX, et al. Phospholipase C delta4 regulates cold sensitivity in mice. J Physiol. 2016;594(13):3609–3628. 27062607 10.1113/JP272321 PMC4929334

[48] Kim AY, Tang Z, Liu Q, et al. Pirt, a phosphoinositide-binding protein, functions as a regulatory subunit of TRPV1. Cell. 2008;133(3):475–485. 18455988 10.1016/j.cell.2008.02.053 PMC2605970

[49] Tang M, Wu GY, Dong XZ, et al. Phosphoinositide interacting regulator of TRP (Pirt) enhances TRPM8 channel activity in vitro via increasing channel conductance. Acta Pharmacol Sin. 2016;37(1):98–104. 26657057 10.1038/aps.2015.110 PMC4722975

[50] Sisco NJ, Helsell CVM, Van Horn WD. Competitive interactions between PIRT, the cold sensing ion channel TRPM8, and PIP2 suggest a mechanism for regulation. Sci Rep. 2019;9(1):14128. 31575973 10.1038/s41598-019-49912-5 PMC6773951

[51] Hilton JK, Salehpour T, Sisco NJ, et al. Phosphoinositide-interacting regulator of TRP (PIRT) has opposing effects on human and mouse TRPM8 ion channels. J Biol Chem. 2018;293(24):9423–9434. 29724821 10.1074/jbc.RA118.003563 PMC6005438

[52] Liu B, Qin F. Functional control of cold- and menthol-sensitive TRPM8 ion channels by phosphatidylinositol 4,5-bisphosphate. J Neurosci. 2005;25(7):1674–1681. 15716403 10.1523/JNEUROSCI.3632-04.2005 PMC6725927

[53] De Petrocellis L, Starowicz K, Moriello AS, et al. Regulation of transient receptor potential channels of melastatin type 8 (TRPM8): effect of cAMP, cannabinoid CB(1) receptors and endovanilloids. Exp Cell Res. 2007;313(9):1911–1920. 17428469 10.1016/j.yexcr.2007.01.008

[54] Linte RM, Ciobanu C, Reid G, et al. Desensitization of cold- and menthol-sensitive rat dorsal root ganglion neurones by inflammatory mediators. Exp Brain Res. 2007;178(1):89–98. 17006682 10.1007/s00221-006-0712-3

[55] Bavencoffe A, Gkika D, Kondratskyi A, et al. The transient receptor potential channel TRPM8 is inhibited via the alpha 2A adrenoreceptor signaling pathway. J Biol Chem. 2010;285(13):9410–9419. 20110357 10.1074/jbc.M109.069377 PMC2843190

[56] Sarria I, Gu J. Menthol response and adaptation in nociceptive-like and nonnociceptive-like neurons: role of protein kinases. Mol Pain. 2010;6:47. 20727164 10.1186/1744-8069-6-47 PMC2936373

[57] Zhang X, Mak S, Li L, et al. Direct inhibition of the cold-activated TRPM8 ion channel by Galphaq. Nat Cell Biol. 2012;14(8):851–858. 22750945 10.1038/ncb2529 PMC3428855

[58] Li L, Zhang X. Differential inhibition of the TRPM8 ion channel by Galphaq and Galpha 11. Channels (Austin). 2013;7(2):115–118. 23334401 10.4161/chan.23466 PMC3667880

[59] Klasen K, Hollatz D, Zielke S, et al. The TRPM8 ion channel comprises direct Gq protein-activating capacity. Pflugers Arch. 2012;463(6):779–797. 22460725 10.1007/s00424-012-1098-7

[60] Liu L, Yudin Y, Nagwekar J, et al. Galphaq sensitizes TRPM8 to inhibition by PI(4,5)P2 depletion upon receptor activation. J Neurosci. 2019;39(31):6067–6080. 10.1523/JNEUROSCI.2304-18.2019 PMC6668199 31127000

[61] Zhang X. Direct Galphaq Gating is the sole mechanism for TRPM8 Inhibition caused by bradykinin receptor activation. Cell Rep. 2019;27(12):3672–83e4. 31216483 10.1016/j.celrep.2019.05.080 PMC6595177

[62] Sharma N, Flaherty K, Lezgiyeva K, et al. The emergence of transcriptional identity in somatosensory neurons. Nature. 2020;577(7790):392–398. 31915380 10.1038/s41586-019-1900-1 PMC7307422

[63] Usoskin D, Furlan A, Islam S, et al. Unbiased classification of sensory neuron types by large-scale single-cell RNA sequencing. Nat Neurosci. 2015;18(1):145–153. 25420068 10.1038/nn.3881

[64] Premkumar LS, Raisinghani M, Pingle SC, et al. Downregulation of transient receptor potential melastatin 8 by protein kinase C-mediated dephosphorylation. J Neurosci. 2005;25(49):11322–11329. 16339027 10.1523/JNEUROSCI.3006-05.2005 PMC6725906

[65] Abe J, Hosokawa H, Sawada Y, et al. Ca2+-dependent PKC activation mediates menthol-induced desensitization of transient receptor potential M8. Neurosci Lett. 2006;397(1–2):140–144. 16380208 10.1016/j.neulet.2005.12.005

[66] Pabbidi MR, Premkumar LS. Role of transient receptor potential channels TRPV1 and TRPM8 in diabetic peripheral neuropathy. J Diab Treat. 2017;2017(4):029. PMC6317870 30613832

[67] Iftinca M, Basso L, Flynn R, et al. Chronic morphine regulates TRPM8 channels via MOR-PKCbeta signaling. Mol Brain. 2020;13(1):61. 32290846 10.1186/s13041-020-00599-0 PMC7155267

[68] Taberner FJ, Lopez-Cordoba A, Fernandez-Ballester G, et al. The region adjacent to the C-end of the inner gate in transient receptor potential melastatin 8 (TRPM8) channels plays a central role in allosteric channel activation. J Biol Chem. 2014;289(41):28579–28594. 25157108 10.1074/jbc.M114.577478 PMC4192508

[69] Li HW, Zhou B, Zhang HH. [TRPM8 mediates PC-12 neuronal cell apoptosis induced by oxygen-glucose deprivation through cAMP-PKA/UCP4 signaling]. Nan Fang Yi Ke Da Xue Xue Bao. 2016;36(9):1265–1270. 27687662

[70] Asuthkar S, Demirkhanyan L, Sun X, et al. The TRPM8 protein is a testosterone receptor: II. Functional evidence for an ionotropic effect of testosterone on TRPM8. J Biol Chem. 2015;290(5):2670–2688. 25480785 10.1074/jbc.M114.610873 PMC4316998

[71] Asuthkar S, Elustondo PA, Demirkhanyan L, et al. The TRPM8 protein is a testosterone receptor: I. Biochemical evidence for direct TRPM8-testosterone interactions. J Biol Chem. 2015;290(5):2659–2669. 25480783 10.1074/jbc.M114.610824 PMC4317017

[72] Asuthkar S, Velpula KK, Elustondo PA, et al. TRPM8 channel as a novel molecular target in androgen-regulated prostate cancer cells. Oncotarget. 2015;6(19):17221–17236. Epub 2015/ 05/20. 25980497 10.18632/oncotarget.3948 PMC4627303

[73] Gkika D, Lolignier S, Grolez GP, et al. Testosterone-androgen receptor: the steroid link inhibiting TRPM8-mediated cold sensitivity. Faseb J. 2020;34(6):7483–7499. 10.1096/fj.201902270R 32277850

[74] Ge MM, Zhou YQ, Tian XB, et al. Src-family protein tyrosine kinases: A promising target for treating chronic pain. Biomed Pharmacothe. 2020;125:110017. 10.1016/j.biopha.2020.110017 32106384

[75] Babes A, Zorzon D, Reid G. Two populations of cold-sensitive neurons in rat dorsal root ganglia and their modulation by nerve growth factor. Eur J Neurosci. 2004;20(9):2276–2282. 15525269 10.1111/j.1460-9568.2004.03695.x

[76] Manolache A, Selescu T, Maier GL, et al. Regulation of TRPM8 channel activity by Src-mediated tyrosine phosphorylation. J Cell Physiol. 2020;235(6):5192–5203. 31729029 10.1002/jcp.29397

[77] Gkika D, Lemonnier L, Shapovalov G, et al. TRP channel-associated factors are a novel protein family that regulates TRPM8 trafficking and activity. J Cell Biol. 2015;208(1):89–107. 25559186 10.1083/jcb.201402076 PMC4284226

[78] Taylor DJR, Hamid SM, Andres AM, et al. Antiviral effects of menthol on Coxsackievirus B. Viruses. 2020;12:4. 10.3390/v12040373 PMC7232514 32231022

[79] Bidaux G, Gordienko D, Shapovalov G, et al. 4TM-TRPM8 channels are new gatekeepers of the ER-mitochondria Ca(2+) transfer. Biochim Biophys Acta Mol Cell Res. 2018;1865(7):981–994. 29678654 10.1016/j.bbamcr.2018.04.007

